# Effects of Dietary Inclusion of Lentil Byproduct on Performance and Oxidative Stability of Eggs in Laying Quail

**DOI:** 10.1155/2014/742987

**Published:** 2014-08-11

**Authors:** Metin Çabuk, Serdar Eratak, Hatice Basmacioğlu Malayoğlu

**Affiliations:** ^1^Department of Poultry Science, Vocational School of Celal Bayar University, Akhisar, 45210 Manisa, Turkey; ^2^Department of Animal Science, Agricultural Faculty of Ege University, Bornova, 35100 Izmir, Turkey

## Abstract

One hundred and sixty-eight 11-week-old laying quails *(Coturnix coturnix japonica)* were fed one of the following three diets: (1) control: basal diet with no lentil *(Lens culinaris L.)* byproduct; (2) inclusion of 10% lentil byproduct; (3) inclusion of 20% lentil byproduct. In the recent years, colour sorting machines are used in order to separate red lentils according to their colours. The goal is to select the items which are discoloured, not as ripe as required, or still with hull even after dehulling of lentil seed. During the sorting, a new byproduct called “sorting byproduct” leftover is obtained. The byproduct is cleaner and is of a higher quality than other lentil byproducts. This experiment was conducted to study the effects of the inclusion of different levels of lentil byproduct on laying quail performance. The experimental treatment included 10% or 20% lentil byproduct in the diet, and this was fed to quails aged between 11 and 22 weeks. The inclusion of 10% and 20% levels of lentil byproduct in the diet significantly increased egg production, but feed intake and feed conversion ratio were not significantly affected. Egg weight decreased significantly following the inclusion of 20% lentil byproduct. The inclusion of lentil byproduct in the diet increased the deposition of yellow yolk pigments and decreased malonaldehyde formation in the yolk.

## 1. Introduction

Leguminous seeds used in human nutrition are processed before being made available for consumption, and during this process various byproducts are generated, such as pea byproduct, lentil byproduct, and sunflower meal. These byproducts are used in animal rations as cheap feed sources. Red lentil's peel is removed and cleaned while it is processed in the factories as edible product. After this process, in the recent years, color sorting machines are used in order to separate harvested food stuffs such as red lentils according to their colors. The goal is the separation of items that are discoloured, not as ripe as required, or still with hull after dehulling lentil seed [1]. During this sorting, a new byproduct called “sorting byproduct” leftover is obtained. This byproduct comprises 2–4% of total processed lentil. It is cleaner and is of a higher quality than other lentil byproducts; however, it is not suitable for human consumption. Although lentil (*Lens culinaris *L.) is primarily used for food, it also provides an excellent protein-rich (22%–29%) feedstuff for domestic livestock. In addition, crop residues and byproducts from the food processing industry can be effectively utilized by livestock, particularly poultry. Lentil byproducts are an excellent source of macronutrients and contain phytochemicals [2] that can be categorized into phenolic acids, soyasaponins, phytic acid, and condensed tannins [3]. Lentil and lentil byproduct exhibit a high total antioxidant capacity, likely related to its high content of condensed tannins [3]. Until recently, phenolic compounds were regarded as nonnutritive compounds and it was reported that excessive content of polyphenols, particularly tannins, may have adverse consequences because of inhibition of the bioavailability of iron and blockage of digestive enzymes in the gastrointestinal tract [4]. Phenolic compounds can also limit the bioavailability of proteins with which they form insoluble complexes in the gastrointestinal tract. Subsequently, the significance of phenolic compounds was gradually recognized and several researches have now reported that phenolics offer many health benefits and are vital to human nutrition [3]. High correlations between phenolic composition and antioxidant activities have been reported by Tsopmo and Muir [5]. Lentil and lentil byproducts with the highest total phenolic content exhibit the highest antioxidant and oxygen radical absorbance capacity [6]. Because no report to date has dealt with the antioxidant effect on eggs by tannins following the dietary inclusion of lentil byproduct, the objectives of this research were to investigate the effect of such dietary inclusion on delayed lipid oxidation on eggs and the performance of laying quail.

## 2. Materials and Methods 

All animal protocols received prior approval of the Institutional Animal Care and Use Committee, and all experiments were performed in accordance with relevant guidelines and regulations. One hundred and sixty-eight 11-week-old laying quails (*Coturnix coturnix japonica*) were fed one of the following three diets over an 11-week period: control: basal diet with no lentil byproduct; inclusion of 10% lentil byproduct; inclusion of 20% lentil byproduct. Each treatment comprised 4 replications with 4 cages (14 quails per cage), amounting to 56 quails per treatment group. A photoperiod of 17 h/day was maintained, and egg production was recorded daily. During the laying period, a random sample of 10 eggs/replicate was collected on 2 consecutive days per week for determination of egg weight. Feed intake was recorded on a weekly basis and feed conversion ratio (FCR) was calculated as the ratio of grams of feed consumed per gram egg weight. Levels of production variables such as feed intake and egg production were adjusted for mortality rates, mortality being recorded daily. All diets were isocaloric and isonitrogenous (in mash form), with water being provided* ad libitum*. At the end of the experiment (22 weeks of age), 24 eggs were collected from each treatment and analyzed for yolk color with a Minolta Chroma Meter CR-300 using the CIE (Commission Internationale de L'Enclairage) scale. L*, a*, and b* values reflect lightness (0 = black, 100 = white), redness (−100 = green, 100 = red), and yellowness (−100 = blue, 100 = yellow) of the samples, respectively. The albumen was removed from the yolk before yolk color evaluation.

**Figure 1:**
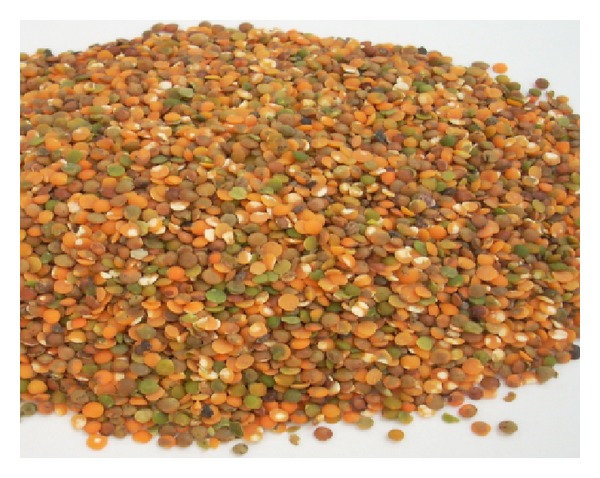
Lentil byproduct used in the experiment.

To investigate the effects of diet on shell eggs lipid oxidation during refrigeration, 10 eggs from each replicate collected at the end of treatment (40 eggs from each dietary treatment) were placed in a refrigerated cabinet at 4°C for analysis of yolk thiobarbituric acid (TBA) content at 0, 7, 14, 21, and 28 days of storage. TBA values were determined as previously described by Cherian et al. [7]. Egg yolk samples (2 g, *n* = 8) were weighed into 50 mL test tubes and 18 mL of 3.86% perchloric acid was added. The samples were homogenized with a vortex, with butylated hydroxytoluene added to each sample during homogenization to control lipid oxidation. The homogenate was then filtered through Whatman 1 filter paper and the filtrate was (2 mL) mixed with 2 mL of 20 mM TBA in distilled water. The filtrate was incubated in a darkened cabinet at room temperature overnight (15–17 h), with absorbance determined at 531 nm. TBA values were expressed as milligrams of malonaldehyde (MDA) per kilogram of yolk. Ten eggs from each replicate were collected at the end of treatment (40 eggs from each dietary treatment) for pH analysis. The eggs (yolk + white) were homogenized and pH was measured with a pH meter. Condensed tannins in the lentil byproduct were assayed in accordance with the butanol-HCl method of Makkar [8]. The experimental diets and lentil byproduct were also analyzed for dry matter, starch, sucrose, crude protein, ether extract, and crude ash using the method of the Association of German Agricultural Analysis and Research Institutes (VDLUFA) [9]. Red lentils' peels are removed and cleaned while they are processed as edible products in factories. After this process, color sorters are machines that are used on the production lines in bulk food processing. The machines separate red lentil by their colors. The goal is the separation of items that are discoloured, not as ripe as required, or still with hull after dehulling lentil seed. During this sorting, a new byproduct called “sorting byproduct” leftover is obtained. The chemical composition of lentil byproduct is shown in Table 1 and Figure 1. The ingredients and chemical composition of the basal diet are presented in Table 2. Data were analyzed using the general linear model procedure of SAS [10], and differences were considered significant at *P* < 0.05.

## 3. Results and Discussion

**Figure 2:**
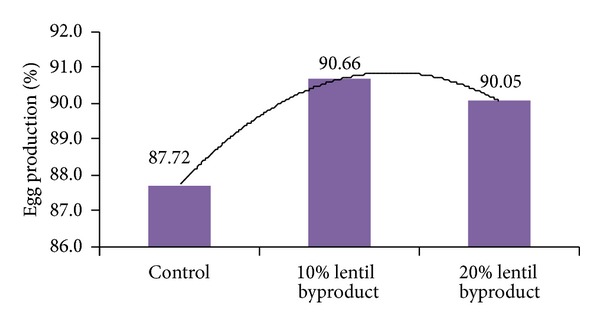
Effect of lentil byproduct on egg production.

**Figure 3:**
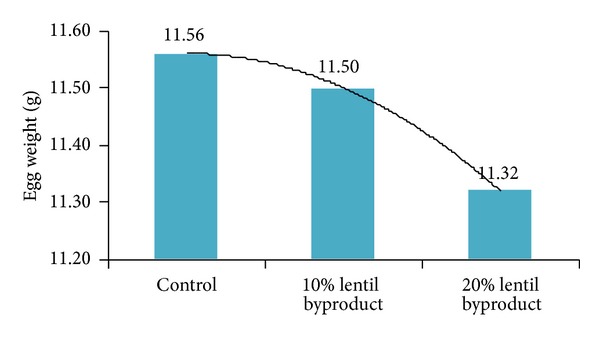
Effect of lentil byproduct on egg weight.

The effects of dietary lentil byproduct on egg production, feed intake, FCR, and egg weight are presented in Table 3 and Figures 2 and 3.

**Figure 4:**
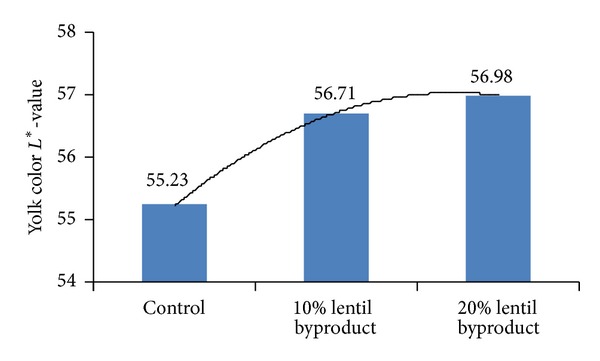
Effect of lentil byproduct on yolk color L* value, L* = 0 denoting dark and values close to L* = 100 denoting light.

**Figure 5:**
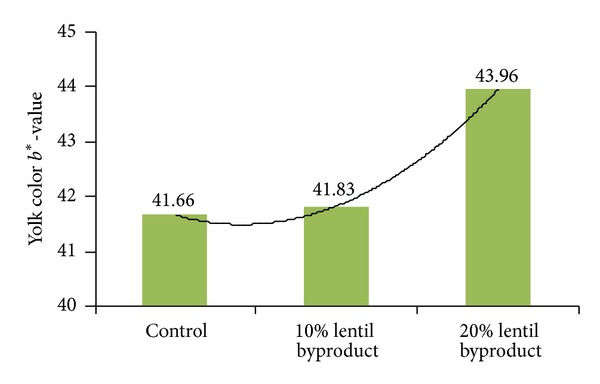
Effect of lentil byproduct on yolk b* value; if b* value minus (−) blue, plus (+) the yellow color.

There were significant effects on egg production and egg weight. The inclusion of 10% and 20% lentil byproducts in the diet increased (*P* < 0.01) hen-day egg production above that of the control diet, with egg production being similar for both diets including lentil byproduct. However, the inclusion of lentil byproduct in excess of 10% had no additional beneficial effect on egg production. In contrast to our results, Kılıçalp and Benli [11] observed that the inclusion of 10% dietary lentil byproduct decreased hen-day egg production. Although the inclusion of lentil byproduct in our study up to a level of 10% had no negative effect on egg weight, this was reduced significantly with the inclusion of 20% dietary lentil byproduct compared with the control diet. In agreement with our results, Kılıçalp and Benli [11] also reported that the inclusion of dietary lentil byproduct in excess of 10% decreased egg weight. In our study, there were no differences (*P* > 0.05) in feed intake and FCR between treatments over the 11-week period. Similar results were reported by Kılıçalp and Benli [11], who reported that the dietary inclusion of lentil byproduct at 20% had no beneficial effect on feed intake. However, in contrast to our findings, these authors reported that the dietary inclusion of lentil byproduct in excess of 10% adversely affected FCR. Significant dietary effects on all yolk color parameters were noted in our study (Table 4 and Figures 4 and 5). Lightness (L*) was increased with the inclusion of dietary lentil byproduct compared with the control diet, but the inclusion of lentil byproduct in excess of 10% had no additional beneficial effect.

**Figure 6:**
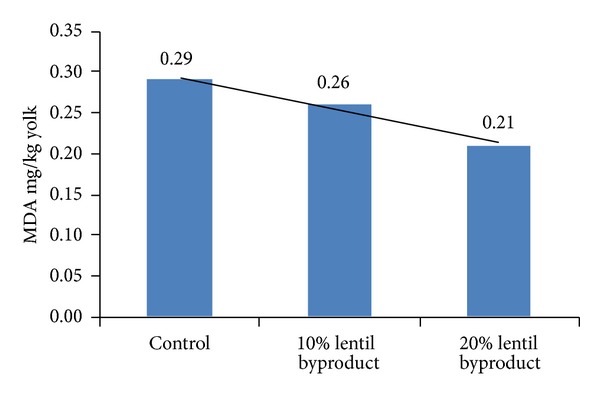
Effect of lentil byproduct on lipid oxidation.

**Figure 7:**
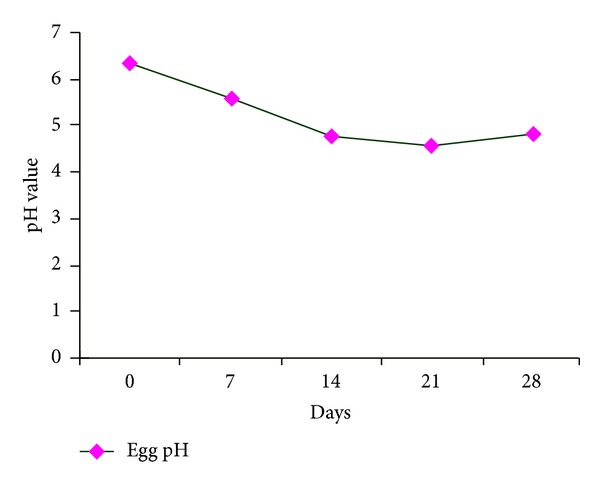
Effect of storage time on egg pH.

The a* (red-green) parameter of yolk was not significantly affected by the dietary inclusion of lentil byproduct; the b* (yellowness) was increased by the inclusion of 20% lentil byproduct, but not by 10%. This result showed that inclusion of lentil byproduct in the diet increased deposition of yellow pigments into the yolk. The effects of these dietary inclusions on the oxidative stability of shell egg measured at different time periods, and on pH, are shown in Table 5 and Figures 6 and 7.

The level of lipid oxidation, as measured by MDA formation, differed (*P* < 0.01) among dietary treatments, but did not change with storage time. MDA formation in eggs decreased with increasing dietary content of lentil byproduct. Total antioxidant capacity and the mechanism of action of tannin in lentil were analyzed in vitro studies. The lentil was shown to have a marked total antioxidant capacity, likely related to its high content of condensed tannins [3, 5, 6]. However, reports are limited on the dietary inclusion of lentil byproduct aimed at increasing the oxidative stability of shell egg. Although egg pH was not affected by dietary treatment in our study, it decreased with longer refrigerated storage time as recorded on days 0, 7, 14, 21, and 28 (*P* < 0.01).

## 4. Conclusion

The inclusion of lentil byproduct in the diet of laying quail was beneficial in terms of increased egg production and deposition of yellow yolk pigments. Moreover, lipid oxidation was reduced in shell egg. Additional research is needed to investigate the antioxidant constituents of tannin in lentil byproduct when deposited in egg yolk.

## Figures and Tables

**Table 1:** Nutrient and tannin contents of lentil byproduct.

Dry matter, %	88.20
Crude protein, %	25.42
Ether extract, %	2.19
Crude cellulose, %	3.48
Crude ash, %	6.11
Starch, %	42.9
Sucrose, %	3.12
Condensed tannin; g/kg, dry matter	4.41
Metabolisable energy, Kcal/kg	2930

**Table 2:** Ingredient and chemical composition of diets used in the study (as fed).

Ingredients (%)	Control	10% lentil byproduct	20% lentil byproduct
Corn	57.22	51.71	45.86
Full fat soybean	16.24	15.77	16.02
Fish meal, (0.65 crude protein)	3.00	3.00	3.00
Soybean meal (0.48 crude protein)	13.00	9.00	4.51
Lentil by product	0	10.0	20.0
Methionine	0.08	0.02	0.06
Monocalcium phosphate	1.13	1.18	1.25
Ground limestone	8.88	8.87	8.85
Salt	0.20	0.20	0.20
Vitamin-mineral premix∗	0.25	0.25	0.25
Total	**100**	**100**	**100**

Composition, % (analyzed)			
Dry matter	90.57	91.80	91.50
Crude protein	17.69	17.78	17.56
Crude cellulose	3.12	2.68	2.34
Crude ash	10.94	11.85	11.98
Ether extract	5.49	4.91	4.89
Starch	43.79	44.67	44.65
Sucrose	2.7	2.5	2.7
Metabolisable energy, Kcal/kg	2929	2921	2917

Calculated composition, %			
Methionine + cysteine	0.74	0.74	0.74
Lysine	1.10	1.09	1.08
Calcium	3.50	3.49	3.50
Available phosphorus	0.41	0.41	0.40

∗Supplied per kg of diet: vitamin A, 12 000 IU; vitamin D3, 3 000 IU; vitamin E, 30 mg; vitamin K3, 5 mg; vitamin B1, 3 mg; vitamin B2, 12 mg; vitamin B6, 4 mg; vitamin B12, 0.03 mg; nicotinamide, 55 mg; calcium-D-pantothenate, 15 mg; folic acid, 2 mg; D-biotin, 0.250 mg; choline chloride, 150 mg; Mn, 80 mg; Fe, 40 mg; Zn, 60 mg; Cu, 5 mg; I, 0.4 mg; Co, 0.1 mg; Se, 0.15 mg.

**Table 3:** The effect of the dietary inclusion of lentil byproduct on egg production, FCR and feed intake, and egg weight of quails.

Parameter	Control	10% lentil byproduct	20% lentil byproduct	Pooled SEM	*P*
Egg production, %	87.72^b^	90.66^a^	90.05^a^	0.52	0.0001
Egg weight, g	11.56^a^	11.50^a^	11.32^b^	0.04	0.0001
Feed intake, g/quail	25.69	25.63	25.22	0.47	0.7464
Feed conversion ratio, g, fed/g, egg	2.53	2.48	2.49	0.04	0.5732

^a,b^Means within row with no common superscripts differ significantly (*P* < 0.05).

SEM: standard error of the mean.

**Table 4:** The effect of the dietary inclusion of lentil byproduct on yolk color.

Groups/parameters	L*-value	a*-value	b*-value
Control	55.23^b^	−1.81	41.66^b^
10% lentil byproduct	56.71^a^	−1.48	41.83^b^
20% lentil byproduct	56.98^a^	−1.76	43.96^a^
Pooled SEM	0.392	0.2934	0.562
*P*	0.0480	0.5118	0.0079

L* = 0 denoting dark and values close to L* = 100 denoting light.

If a* value minus (−) green, plus (+) the red color.

If b* value minus (−) blue, plus (+) the yellow color.

^a,b^Means within column with no common superscripts differ significantly (*P* < 0.05).

SEM: standard error of the mean.

**Table 5:** Effects of lentil byproduct on lipid oxidation (mg MDA/kg yolk) and pH of quail eggs.

		mg MDA/kg egg yolk	pH
Treatment groups	Control	0.29^a^	5.14
	10% lentil byproduct	0.26^b^	5.30
	20% lentil byproduct	0.21^c^	5.20
Pooled SEM		0.008	0.056

Storage time, day	0	0.27	6.35^d^
	7	0.24	5.56^c^
	14	0.25	4.78^b^
	21	0.26	4.54^a^
	28	0.25	4.81^b^
Pooled SEM		0.010	0.073

Probability			
Treatment		0.0001	0.1170
Storage time		0.138	0.0001
